# Occurrence of cancer at multiple sites: Towards distinguishing multigenesis from metastasis

**DOI:** 10.1186/1745-6150-3-14

**Published:** 2008-04-11

**Authors:** Wei-Kang Zhang, Chun Zhang, Jing J Zhang, Shi V Liu

**Affiliations:** 1 Department of General Surgery, Union Hospital, Huazhong Science and Technology University, Wuhan, China; 2 Institute of Hematology, Union Hospital, Huazhong Science and Technology University, Wuhan, China; 3 Waverly Primary Care, Cary, USA; 4 Eagle Institute of Molecular Medicine, Apex, USA

## Abstract

**Background:**

Occurrence of tumors at multiple sites is a hallmark of malignant cancers and contributes to the high mortality of cancers. The formation of multi-site cancers (MSCs) has conventionally been regarded as a result of hematogenous metastasis. However, some MSCs may appear as unusual in the sense of vascular dissemination pattern and therefore be explained by alternative metastasis models or even by non-metastatic independent formation mechanisms.

**Results:**

Through literature review and incorporation of recent advance in understanding aging and development, we identified two alternative mechanisms for the independent formation of MSCs: 1) formation of separate tumors from cancer-initiating cells (CICs) mutated at an early stage of development and then diverging as to their physical locations upon further development, 2) formation of separate tumors from different CICs that contain mutations in some convergent ways. Either of these processes does not require long-distance migration and/or vascular dissemination of cancer cells from a primary site to a secondary site. Thus, we classify the formation of these MSCs from indigenous CICs (iCICs) into a new mechanistic category of tumor formation – multigenesis.

**Conclusion:**

A multigenesis view on multi-site cancer (MSCs) may offer explanations for some "unusual metastasis" and has important implications for designing expanded strategies for the diagnosis and treatment of cancers.

**Reviewers:**

This article was reviewed by Carlo C. Maley nominated by Laura F. Landweber and Razvan T. Radulescu nominated by David R. Kaplan. For the full reviews, please go to the Reviewers' comments section.

## Background

Cancer often appears at multiple sites of the same patient and such malignancy is associated with the high mortality of cancer. Except for some systemic cancers such as leukemia [1] and cancers with clear direct anatomic linkages such as lymphoma [2-4], the conventional view on the multi-organ occurrence of cancer is that cancer cells leave a primary tumor site and arrive at one or more different site(s) to form additional secondary tumor(s) via a comprehensive cascading process called metastasis [5,6]. This classical cascade includes local migration and invasion, dissemination through vascular system, entry and colonization in new environment, and finally proliferation, such that they out-compete indigenous cells [7].

However, as the last frontier of cancer research, metastasis is still poorly understood despite over a century of intensive research [8-10]. Towards a better understanding, investigators in the field of multi-site cancer research currently focus on the properties of cancer cells that confer upon them a metastatic capability [11-16].

More recently and importantly, a process preceding cellular metastasis has been proposed and termed "oncoprotein metastasis [17]." This concept is embedded in the novel "peptide string theory [18-20]" which in turn constitutes an extension of a new physics-based understanding of life: *particle biology *[20-22].

Moreover, many reports have shown unusual "metastasis" of cancers which occur at some remote locations that appear hard to explain by any direct vascular linkage [23-26]. Thus, in order to have a full understanding of multi-site cancers, several possible mechanistic aspects for the cancer formation in multi-sites need to be addressed one which is considered in this paper.

## Hypothesis and rationales

We propose that multigenesis – the formation of cancer in multiple sites by indigenous cancer initiating cells (iCICs) – may be a basis for some multi-site cancers (MSCs). Our hypothesis may be argued on the following theoretical ground and clinical evidence:

1. From a physicochemical point of view, it is possible that mutagenic factor(s) can strike multiple cells at different body sites and thus independently causes same or different mutations in different cells in different sites. The presence of multiple mutations in a typical cancer has been proven by recent genomic screenings in some cancers [27]. Although it is unclear which of these mutations are the main cause for the various cancers it is clear that the presence of the many different mutations in the different body sites could provide a theoretical basis for the formation of independent primary cancer at the multiple sites.

In the past a "clonal evolution" theory has been used for explaining the differences observed between "primary" and "secondary" cancers [28-30]. However, these "site" differences of the "same" cancers may be a false "convergence" as they may represent truly different cancers derived from separate cancer-initiating cells. This multigenesis of cancer-initiating cells (CICs) may also explain why there was even some "unexpectedly high genetic divergence" in "minimal residual cancer [31]."

We should also point out that the parallel mutagenesis for multigenesis of anatomically separate cancers is different from "the parallel evolution model" for metastasis [32]. In the latter model, it is hypothesized that the differences found between primary and secondary tumors [33], especially those solid tumors [34], are results of parallel but different evolution of the same cancers cells whose metastasis have occurred earlier [35].

Since the term "mutation" could conceivably be extended to include not just the mutation in the protein-encoding DNA sequence but also a modification in the epigenetic status of the DNA, the structure of a chromosome, and the conformation of a protein, the latter of which may translate into a "conformational mutation" correlating with a loss of function [36], it is possible that at least one of these distinct types of mutations could independently occur in cells at multiple sites. The sharing of a common underlying mutation mechanism may yield an apparent *"convergent" *phenotype to the separate tumors derived from a common type of mutation. But these independent cancers *would be *by no means a result of any "metastasis" because they do not share a common ancestor cancer-initiating cell (CIC).

2. From a development perspective [37], it is very likely a cell mutated earlier in embryogenesis may contribute to the formation of multi-organ cancers when its offspring cells inheriting the same mutation migrate to different sites to form other tissues in the different organs. In other words, a "jackpot" (gestational) mutation [38] early in development may produce mutant cells that end up in different organs. This formation of multi-site cancer due to this developmental separation of mutated cells is different from the conventional metastasis because the mutated cells are truly indigenous to the various organs at the time of their formation. As a matter of fact, many stem cell cancers [39-41] may be called multigenesis cancers rather than metastasis cancers.

We noticed that the "same gene model" actually holds this same view that genetic alterations can be acquired early in carcinogenesis [42]. But that model has been mainly used for explaining the resemblance of gene expression signature [43,44] or genetic alterations [45] between primary and "secondary" cancers under a predetermined assumption of "metastasis" [42,45]. However, as we argued in the first point, the same gene can be mutated in anatomically separate cells, leading to the multigenesis of different cancers that does not share any direct vascular connection.

**Figure 1:**
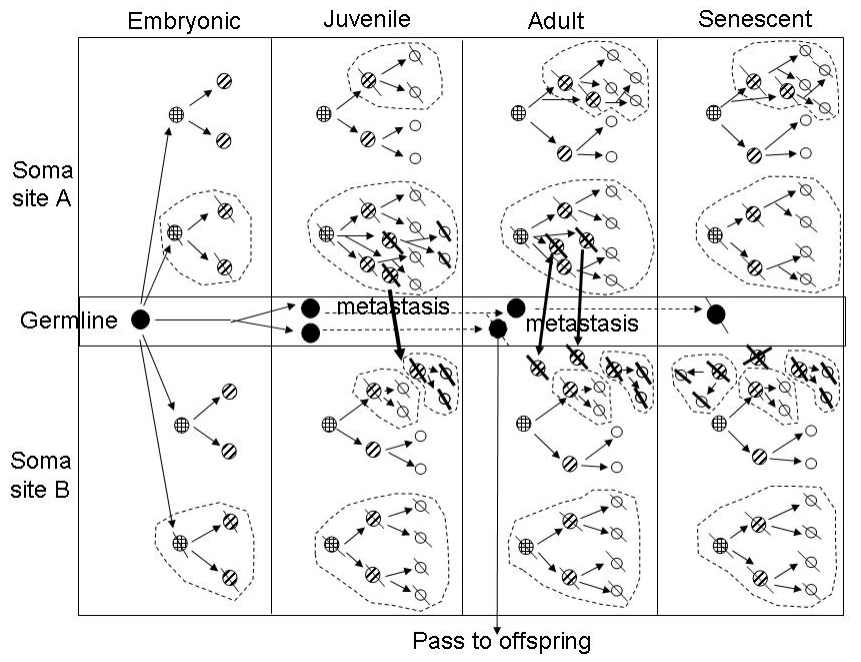
**Schematic representation of multi-site cancer formation by multigenesis and how to distinguish multigenesis from metastasis**. Germ (G), stem (S), progenitor (P) and terminal (T) cells are represented by solid, crossed, slashed and hollow circles, respectively. Subsequent to a mutation that changes a normal cell (without slash across the circle) into a cancer cell (with a slash across the circle), depending on the nature of cancer-initiating cells (CICs), the clones (encircled by a dashed polygon) may contain different compositions of cells at different differentiation stages. There is also some intrinsic cell age heterogeneity in all clones which is indicated in one way by showing the generation succession with solid arrow lines. The continued existence of the mother cell over the different development stages is indicated by dashed arrow lines as shown for germline cells. A mutation in a germ cell may pass into offspring if it occurs at any pre-senescent stage. The distinction between a cancer cell translocated from a primary tumor site (site A) to a secondary site (site B) and a cancer cell originated from an indigenous cell in the same site (site B) may be made by determining a difference in mutations and/or in cell age even if the cells are otherwise genetically identical. Alternatively, an artificial marker may be introduced into offspring cells of the CICs during their reproduction in a primary cancer site so that these cells are distinct (indicated by a thick slash across the circle) from the rest. This experimental approach may offer a way to distinguish metastatic cancer cells from indigenous cancer cells even if they share the same mutations due to a convergence in the mechanisms of carcinogenesis. The growth of tumors at the different sites may be influenced by the environmental conditions at the different sites so that differential tumor growth rates can be seen even for the same CICs. In addition, metastatic cancer cells may have different fates at the different "soils"; some may establish and proliferate while some may die (as indicated by double heavy slashes). These complexities of multi-site cancers are selectively represented in the diagrams, too.

3. From an aging perspective [46-48], it is very likely the same aging process may result in genetic and/or epigenetic damages to DNA or other molecules in cells located in the different parts of the body which then contribute to the independent origin of CICs and thus non-metastatic MSCs [37,49,50]. This parallelism in cell lineage development, in similarity to the parallelism observed in evolution [51,52], predict that a common aging-mechanism-based carcinogenesis may explain not only why cancers often occur at the older ages but also why older people tend to have multiple cancers [50,53]. The detailed hypothesis of a linkage between DNA molecule aging and cell aging [46] and the existence of such aging axis from molecule to cell and to multicellular organism [37] may provide a foundation for drawing a roadmap for not only normal development but also some abnormal processes [50]. To illustrate how environmental factors can disturb the living processes in various ways and thus contribute to the formation of multi-site cancers, we schematically depict some key aspects of carcinogenesis over a representative life span (Fig. 1).

4. From a pathological point of view, "primary" tumors of "metastatic cancers" are often identified at the easily observable or life-threatening sites while "secondary" tumors of "metastatic cancers" are often detected at sites that are either difficult to be discovered or not critical to influence the normal life. It is logically hard to argue why the primary occurrence of carcinogenesis often happens in those easily observable and/or life-threatening sites. This argument is even more difficult to be accepted when the carcinogenic cells in the multiple organs are of the same tissue type and even the same cell type. However, it would make more sense to explain the apparent time-lagging between these "primary" and "secondary tumors as a result of either the different growth rates of the same cancer cells in the different sites or the different detection rates of the same tumor at the different sites or a combination of both factors. Thus, the growth of tumors at the different sites and the understanding of cancer dormancy or latency [54] is not only related with the difference in "soils" but also the difference in "seeds".

5. From recent understanding of genome organization it is clear that genomes are composed of various modules [55,56]. Theoretically, independent mutations can happen by a common mechanism on the same type of DNA module that contributes to different genes or genes' regulatory elements. As a matter of fact, more and more evidences are accumulating to support this mechanism of multi-gene and multi-site mutations/alterations in genome and the subsequent cancer formation [57-60]. Thus, rather starting a series of primary-secondary cancers from one mutation in one cell, multi-site cancers can originate from multiple mutations/alterations on the same genes/genome locations or the same module in different genes. Indeed, recent studies have shown that the same transformation in different cell types leads to distinct tumor phenotypes [61]. Moreover, recent studies have also shown the existence of common mutated genes between breast and colorectal cancers [27,62]. This new information may offer an explanation for the increased prevalence of colorectal cancer in breast cancer patients [63-73] that was observed much earlier [74-79]. Better understanding of the genetics of colorectal and breast cancer [80] and the developmental lineage of the cancer cells in these two major cancers may offer some insight on the true mechanism of the formation of these often related prevalent multi-site cancers.

6. From clinical observations, we have noticed many reports of so-called unusual "metastasis" of cancers. For example, there have been some report of multi-site cancers buried in solid tissue mass but the other easy-to-spread sites often lack such cancer [23-26]. Interestingly, despite the "unusual" nature of these multi-site cancers, the pairing between the "primary" and the "secondary" tumors in some of these rare "metastasis" cases is very consistent [81,82].

7. Some studies have shown that the locations of distant secondary tumors in many clinical cancers and animal tumors are nonrandom, and their distributions cannot be explained by simple anatomical or mechanical hypotheses based on the simple lodgment or trapping of tumor cell emboli in the first capillary bed encountered [83]. These observations were used for the argument that the unique properties of particular tumor cells ('seeds') and the different characteristics of each organ microenvironment ('soil') collectively determine the organ preference of *metastasis *[83], without, however, advancing additional mechanistic models.

8. Some earlier studies actually have provided evidence that the occurrence of some multiple tumors (MTs) is not due to migration of tumor cells because the tumor cells in different sites are not clonally related [84,85] based on the use of some "clonal" markers [86,87]. However, the use of identity or similarity in "clonal" markers as the only differentiation criterion for distinguishing clonally linked or independent tumor/cancer formation may be misleading. This is because similarity is not always the result of a common origin. That notion is based on the statistical assertion that highly similar patterns in "clonal" markers are unlikely when the mutation is random. However, the very possible truth is that mutation is not random at all. Thus, it is formally possible that the same or similar mutation can happen in multiple cells and yield CICs with similar or even identical "clonal" markers. Thus, the incidence of MPTs or MPCs might have been underestimated by the "clonal" marker in these analyses.

The occurrence of some (anatomically adjacent) multiple primary tumors (MPTs) [88] or multiple primary cancers (MPCs) [89,90] have been explained by a field cancerisation theory [91,92]. In this theory, the initial mutagenesis happens not just in one cell but a whole tract of tissue – a "field". These mutated progenitor cells then spread and cause independent tumors [93]. Thus, field cancerisation could alternatively be interpreted as local multigenesis. By extension, multigenesis should also be possible in distant and separated "fields", thus contributing to the formation of MPTs or MPCs bearing no anatomical relationship.

9. Therapies aimed at eradicating metastasis cancer cells often do not change much the progress of the cancer and the life expectancy of cancer patients [94,95]. This may indicate that cutting off the vascular "connections" among primary and "secondary" tumors may not be sufficient for treating all the "secondary" tumors. Most likely, some of the so-called "secondary" tumors are independent primary tumors which may contain either dormant cancer cells that resist systematic cancer therapy or active cancer cells which escape the killing due to that fact that their reproduction time is outside the treatment window [96,97].

## Implications and impact

We should emphasize that our proposal of a multigenesis mechanism for the occurrence of multi-site cancer does not dispute the occurrence of metastasis which may be even the prevalent cause for many of the multi-site cancers [98-100]. In some cases, the multi-site cancers in a patient may reflect the outcomes of both processes [85]. However, embracing a multigenesis view of multi-site cancer formation may provide additional insights into a comprehensive understanding of the cancer biology and thorough guidance on cancer therapy.

If multigenesis is also a mechanism for the formation of multi-site cancer, then a more proactive diagnosis screening should be implemented even if the incidentally found "primary" cancer is in the very early stage. However, instead of random search, these earlier and broader scope screening may be focused more on the tissues that share the same lineages with the cancer cells in the already found "primary" site.

In the past arguments on metastasis cancers or multiple primary cancers have been focused on whether the mutations in the cancer cells of the different sites are similar or not. These arguments are based on a rationale that random mutation rarely leads to the identical or highly similar mutations in different cells. While these arguments may be true, a better and more definite criterion in separating metastatic cancer from multigenesis cancer may be the distinction of the age of the cancer-initiating cells in each sites. If the CICs at the two different sites have the same or very similar old chronological age then they are more likely originated from independent mutations. By "old" we mean that the CICs have lived longer time than the first discovery of even the primary tumor.

If the multi-site cancers in a patient are originated with multigenesis, the treatment should not just be focused on the first found "primary" cancer site at the later stages or at the easy to be seen location but also be proactively extended into other likely primary sites. On the other hand, the application of cancer treatment need not to be carried out via any systemic way so that more normal reproducing cells could be saved from the non-discriminative killing by the current cancer treatment options. As a matter of fact, studies have shown that systematic treatment damage immune cells in addition to some cancer cells and thus make the patients weaker in dealing with the spared cancers cells once these cancer cells come back to life or proliferation [96,97].

Ideally, highly-targeted cancer drugs should be developed that will be effective only at those proliferating cells bearing the cancer biomarker(s). Certainly, different cancer cells originally from different points of cell lineage formation may have their different biomarkers and thus even cancer drugs themselves should be tailored for the different types of cancers [101].

Yet, the development of cancer is not just a "seed" problem but also a "soil" challenge [8,102,103]. Given that cancer "seeds" could be formed through multiple routes [104,105] and come in as a result of some early spread [106], an effective cancer therapy should also include preventing the "soil"-more precisely, morphologically normal, yet likely premalignant cells-from undergoing oncoprotein-driven malignant transformation [17,107-109].

On the other hand, even some bad "seeds" could conceivably be "corrected" as some (cancer) stem cells appear to be highly moldable and, as such, could be artificially re-directed into some normal development [110-112].

Furthermore, inducing a good soil should not only curtail the growth of bad seeds but also prevent their recurrence [113]. But such good "soils" are difficult to maintain when current non-discriminating systemic cancer therapy destroys many normal cells, too. Thus, overcoming the non-discriminating nature of such classical cancer therapy is a high priority in improving the treatment effectiveness.

In cancer research, cancer biology should be linked more closely with developmental biology so that the non-metastatic linkages between many "unusual" multi-organ cancers can be found by their underlying cell lineage links in development. With this scientific insight and with the power of genomic and other omic technologies [114], biomarkers for the different series of multi-site cancers may be found and used for early detection of multi-site cancers [115,116]. However, cautions should be exerted in differentiating biomarkers for metastasis and biomarkers for multigenesis.

## Conclusion

Multi-site occurrence is the hallmark of the high-grade malignancy cancer and often constitutes the final deadly blow to the patient's life. While metastasis is definitely true for the occurrences of many multi-site cancers [98-100], alternative mechanisms may also exist and deserve attention. The multigenesis hypothesis proposed in this paper may explain some "unusual" occurrences of multi-site cancers. When this perspective on cancer is combined with increasing knowledge of cell lineage formation in hierarchical multicellular organisms [37] more precise predictions on the likely occurrence of multi-site cancers could possibly be made. This multigenesis view of cancer should also lead to an expanded perspective on cancer therapy which may result in the development of more selective medicines that are not only cancer subtype-specific and thus more effective but also less damaging to non-cancer cells.

## Competing interests

The author(s) declare that they have no competing interests.

## Authors' contributions

SVL laid a conceptual foundation for this hypothesis. SVL, WZ, CZ and JJZ participated in the content development of this manuscript. SVL is the principal writer of this manuscript. WZ, CZ and JJZ participated in the writing of the manuscript.

## Reviewers' comments (according to the time sequence of agreeing on performing the review)

### Reviewer 1: Cornelis J. M. Melief, Leiden University Medical Center, Netherlands

This reviewer provided no comment for publication.

### Reviewer 2: Carlo C. Maley, The Wistar Institute, Philadelphia, Pennsylvania, USA

In most of cancer biology, the appearance of multiple neoplasms in different sites is assumed to have been generated by a primary tumor that has metastasized to secondary sites. Zhang et al. have proposed two alternative hypotheses: 1) A "jackpot" (gestational) mutation [38] early in development may produce mutant cells that end up in different organs. 2) Coincidental, convergent but independent evolution of neoplasms at separate sites. Zhang et al. do not claim that these hypotheses explain all or even most cases of multi-site neoplasms, but may explain some "unusual metastasis" cases, especially those in which neither the lymph system nor the vasculature can easily explain how metastatic cells might travel from one site to the other. The unusual metastases are the key to the author's arguments and should be expanded upon (there must be other cases aside from the intracardiac cases mentioned).

These hypotheses are interesting and may be correct, but there is more evidence that should be marshaled in order to convince the community. The two hypotheses make testable predictions, some of which may be addressed through the literature. If neoplasms derive from a jackpot mutation in development then there should be some constraints on which sites would share the mutant clone due to cell lineage fates. In the cases that cannot easily be explained by metastasis, are the sites more often related by development than would be expected by chance? (they authors should review the limited literature on gestational mutations in cancer [38]). If the mutant happened early enough in development to be found in multiple sites, then that clone would probably expand to a fairly large size in both organs, just by normal development (let alone neoplastic clonal expansion). So we would expect to find a large, probably pre-cancerous, patch in both sites. Hereditary cancer syndromes are extreme cases of this hypothesis. Retinoblastoma can show up independently in both eyes, but only in children that have inherited an inactive allele of Rb [39].

The convergent evolution hypothesis should also be easy to study because genotyping of the independent sites should reveal if they are clonally related. While some of the same genes might be activated/inactivated in independent tumors, it is highly unlikely that the exact same point mutation, or the boundaries would be the same on a region of loss of heterozygosity (LOH), deletion or amplification between independent neoplasms. Mitochondrial mutations, X-inactivation and microsatellite shifts, and even cytokeratins [31] have also been used to test clonality. A number of studies have looked at the relationship between primary tumors and (putative) metastases and found evidence of clonality [30,45]. I do not know if this has ever been done on "unusual metastases." If the authors have access to an unusual metastasis, it wold only take 3 SNP arrays (normal and the two sites) to determine if the tumors carry the same or independent LOH lesions. Another prediction of the convergent evolution hypothesis is that the same mutations are selectively advantageous to a clone in the two different sites. One could imagine testing this in an orthotopic xenograft model in which cells of the correct type and with the mutation are injected into the appropriate organ and their fate is tracked. Similarly, there are now inducible genetic models in which a minority of cells in an organ may be genetically manipulated.

#### Authors' response

This is a very careful and helpful review. We have addressed the issues raised by making significant changes and enhancements which include:

"In the past a "clonal evolution" theory has been used for explaining the differences observed between "primary" and "secondary" cancers [28-30]. However, these "site" differences of the "same" cancers may be a false "convergence" as they may represent truly different cancers derived from separate cancer-initiating cells. This multigenesis of cancer-initiating cells (CICs) may also explain why there was even some "unexpectedly high genetic divergence" in "minimal residual cancer" [31].

We should also point out that the parallel mutagenesis for multigenesis of anatomically separate cancers is different from "the parallel evolution model" for metastasis [32]. In the latter model, it is hypothesized that the differences found between primary and secondary tumors [33], especially those solid tumors [34], are results of parallel but different evolution of the same cancers cells whose metastasis have occurred earlier [35]."

"We noticed that the "same gene model" actually holds this same view that genetic alterations can be acquired early in carcinogenesis [42]. But that model has been mainly used for explaining the resemblance of gene expression signature [43,44] or genetic alterations [45] between primary and "secondary" cancers under a predetermined assumption of "metastasis" [42,45]. However, as we argued in the first point, the same gene can be mutated in anatomically separate cells, leading to the multigenesis of different cancers that does not share any direct vascular connection."

"In the past arguments on metastasis cancers or multiple primary cancers have been focused on whether the mutations in the cancer cells of the different sites are similar or not. These arguments are based on a rationale that random mutation rarely leads to the identical or highly similar mutations in different cells. While these arguments may be true, a better and more definite criterion in separating metastatic cancer from multigenesis cancer may be the distinction of the age of the cancer-initiating cells in each site. If the CICs at the two different sites have the same or very similar old chronological age then they are more likely originated from independent mutations. By "old" we mean that the CICs have lived longer time than the first discovery of even the primary tumor. "

The publications referred by this peer reviewer are integrated into this revision and thus we added the citation numbers in the above review accordingly.

### Reviewer 3: Razvan T. Radulescu, Molecular Concepts Research (MCR), Munich, Germany

In their theoretical study entitled "Occurrence of cancer at multiple sites: Metastasis versus multigenesis?" and submitted to *Biology Direct*, Zhang *et al*. propose that *de novo *initiation of cancer in multiple sites may actually account for those clinical cases in which metastasis to "unusual" sites has been ascertained. Yet, one should caution that, as already revealed by the seminal work of Garth Nicolson and associates (cf. e.g. *Cancer Metastasis Rev*. 1988; 7: 143–188), metastatic distributions cannot be explained just by anatomical or mechanical considerations based on the trapping of tumor cells in the first capillary bed along their migratory route, therefore ruling in that "unusual" sites can still be compatible with the process of metastasis.

Nevertheless, the above hypothesis by Zhang *et al*. would have merit to be published provided that it was revised along the following lines. Rather than replacing a valid concept by another, the presented arguments are better suited to explain the well-known, but still unclear multigenesis phenomenon *per se*, specifically those rare, yet significant clinical cases in which a multiple occurrence of carcinomas can be observed in the same tissue or yet in distinct tissues, the so-called multiple primary tumors (MPT).

Since these (clonally distinct, cf. e.g. the *Cancer *reports by Monique van Oijen *et al*., 2000; 88: 884–893 and by Winand Dinjens *et al*., 2003; 97: 1766–1774) neoplasias are most likely induced by adverse/toxic environmental influences (such as cigarette smoke or distinct carcinogenic chemicals) and, moreover, their occurrence may be precipitated by progressive aging, the published hypothesis by Shi V. Liu linking the possibility of an asymmetric segregation of DNA strands during DNA replication (whereby the retained DNA strand in the aging cell accumulates an increasing amount of epigenetic modifications as a result of various environmental exposures) with both cancer and senescence- to which the manuscript by Zhang *et al*. refers on page 2-ought to be described in further detail as a potentially important mechanism accounting for the independent occurrence of primary neoplasias at multiple sites. In this context, an **explanatory diagram **would be helpful.

Moreover, the title of the paper should be amended accordingly, e.g. to read "Occurrence of cancer at multiple sites: Distinguishing multigenesis from metastasis", the word "better" deleted from the background and conclusion sections of the abstract on page 1 and the abstract revised such as to reflect the above suggested shift in conceptual focus on providing a mechanistic basis for the occurrence of MPTs.

Finally, I am aware of the fact that the currently prevailing opinion in cancer therapeutics is to molecularly target individual tumors while sparing (morphologically) normal cells. This is reflected by the following passage in the manuscript by Zhang *et al*.: "If multigenesis is the cause for a multi-site cancer, the application of cancer treatment need not to be carried out via any systemic way but could be made at specific site(s) so that more normal reproducing cells could be saved from the non-discriminative killing by the current cancer treatment options. More ideally, highly-targeted cancer drugs may be developed that will be effective only at those proliferating cells bearing the cancer biomarker(s). Certainly, different cancer cells originally from different points of cell lineage formation may have their different biomarkers and thus even cancer drugs themselves should be tailored for the different types of cancers [64]."

By contrast, I have developed a novel concept over the past years (cf. *Logical Biol*. 2005; 5: 17–29 and 87–88 as well as *arXiv*:0711.4743) according to which, similar to the natural interferon-based protection of non-infected cells during a viral infection, new anti-cancer drugs should not only be directed against neoplastic cells, but also be designed such as to equally prevent normal cells from being transformed and thus ultimately to avert metastasis including what I coined as "oncoprotein metastasis" (cf. *arXiv*:0712.2981 and *Proc. Natl. Acad. Sci. USA *2008, doi/10.1073/pnas.0712232105) or yet, as I specify here, the emergence of MPTs. Towards accelerating future progress in this area, it may therefore be fruitful if Zhang *et al*. touched upon the existence and potential of this published alternative view on treating cancer in their revision accordingly.

#### Authors' response

This is a very solid and constructive review. We have addressed the issues raised by making significant changes and enhancements in the revised version. The major changes made in this revision include:

"More recently and importantly, a process preceding cellular metastasis has been proposed and termed "oncoprotein metastasis"[17]. This concept is embedded in the novel "peptide string theory" [18-20] which in turn constitutes an extension of a new physics-based understanding of life: *particle biology *[20-22].

Moreover, many reports have shown unusual "metastasis" of cancers which occur at some remote locations that appear hard to explain by any direct vascular linkage [23-26]. Thus, in order to have a full understanding of multi-site cancers, several possible mechanistic aspects for the cancer formation in multi-sites need to be addressed one which is considered in this paper."

"Since the term "mutation" could conceivably be extended to include not just the mutation in the protein-encoding DNA sequence but also a modification in the epigenetic status of the DNA, the structure of a chromosome, and the conformation of a protein, the latter of which may translate into a "conformational mutation" correlating with a loss of function [36], it is possible that at least one of these distinct types of mutations could independently occur in cells at multiple sites. The sharing of a common underlying mutation mechanism may yield an apparent *"convergent" *phenotype to the separate tumors derived from a common type of mutation. But these independent cancers *would be *by no means a result of any "metastasis" because they do not share a common ancestor cancer-initiating cell (CIC)."

"The detailed hypothesis of a linkage between DNA molecule aging and cell aging [46] and the existence of such aging axis from molecule to cell and to multicellular organism [37] may provide a foundation for drawing a roadmap for not only normal development but also some abnormal processes [50]. To illustrate how environmental factors can disturb the living processes in various ways and thus contribute to the formation of multi-site cancers, we schematically depict some key aspects of carcinogenesis over a representative life span (Fig. 1)."

"Some studies have shown that the locations of distant secondary tumors in many clinical cancers and animal tumors are nonrandom, and their distributions cannot be explained by simple anatomical or mechanical hypotheses based on the simple lodgment or trapping of tumor cell emboli in the first capillary bed encountered [83]. These observations were used for the argument that the unique properties of particular tumor cells ('seeds') and the different characteristics of each organ microenvironment ('soil') collectively determine the organ preference of *metastasis *[83], without, however, advancing additional mechanistic models.

Some earlier studies actually have provided evidence that the occurrence of some multiple tumors (MTs) is not due to migration of tumor cells because the tumor cells in different sites are not clonally related [84,85] based on the use of some "clonal" markers [86,87]. However, the use of identity or similarity in "clonal" markers as the only differentiation criterion for distinguishing clonally linked or independent tumor/cancer formation may be misleading. This is because similarity is not always the result of a common origin. That notion is based on the statistical assertion that highly similar patterns in "clonal" markers are unlikely when the mutation is random. However, the very possible truth is that mutation is not random at all. Thus, it is formally possible that the same or similar mutation can happen in multiple cells and yield CICs with similar or even identical "clonal" markers. Thus, the incidence of MPTs or MPCs might have been underestimated by the "clonal" marker these analyses.

The occurrence of some (anatomically adjacent) multiple primary tumors (MPTs) [88] or multiple primary cancers (MPCs) [89,90] have been explained by a field cancerisation theory [91,92]. In this theory, the initial mutagenesis happens not just in one cell but a whole tract of tissue – a "field". These mutated progenitor cells then spread and cause independent tumors [93]. Thus, field cancerisation could alternatively be interpreted as local multigenesis. By extension, multigenesis should also be possible in distant and separated "fields", thus contributing to the formation of MPTs or MPCs bearing no anatomical relationship."

"Yet, the development of cancer is not just a "seed" problem but also a "soil" challenge [8,102,103]. Given that cancer "seeds" could be formed through multiple routes [104,105] and come in as a result of some early spread [106], an effective cancer therapy should also include preventing the "soil"-more precisely, morphologically normal, yet likely premalignant cells-from undergoing oncoprotein-driven malignant transformation [17,107-109]."

We have changed the title and revised the abstract by taking into this reviewer's suggestions. We also added a figure to schematically show the occurrence of multi-site cancers by multigenesis and how it can be distinguished from metastasis.

## References

[B1] Barabe F, Kennedy JA, Hope KJ, Dick JE (2007). Modeling the initiation and progression of human acute leukemia in mice. Science.

[B2] Levy O, Deangelis LM, Filippa DA, Panageas KS, Abrey LE (2008). Bcl-6 predicts improved prognosis in primary central nervous system lymphoma. Cancer.

[B3] Panageas KS, Elkin EB, Ben-Porat L, Deangelis LM, Abrey LE (2007). Patterns of treatment in older adults with primary central nervous system lymphoma. Cancer.

[B4] Moore MA (2004). Commentary: the role of cell migration in the ontogeny of the lymphoid system. Stem Cells Dev.

[B5] Gupta GP, Massague J (2006). Cancer metastasis: building a framework. Cell.

[B6] Marx J (2001). Cancer research. New insights into metastasis. Science.

[B7] Sahai E (2007). Illuminating the metastatic process. Nat Rev Cancer.

[B8] Fidler IJ (2003). The pathogenesis of cancer metastasis: the 'seed and soil' hypothesis revisited. Nat Rev Cancer.

[B9] Ma L, Teruya-Feldstein J, Weinberg RA (2007). Tumour invasion and metastasis initiated by microRNA-10b in breast cancer. Nature.

[B10] Ramaswamy S, Ross KN, Lander ES, Golub TR (2003). A molecular signature of metastasis in primary solid tumors. Nat Genet.

[B11] Dalerba P, Clarke MF (2007). Cancer stem cells and tumor metastasis: first steps into uncharted territory. Cell Stem Cell.

[B12] Li F, Tiede B, Massague J, Kang Y (2007). Beyond tumorigenesis: cancer stem cells in metastasis. Cell Res.

[B13] Karnoub AE, Dash AB, Vo AP, Sullivan A, Brooks MW, Bell GW, Richardson AL, Polyak K, Tubo R, Weinberg RA (2007). Mesenchymal stem cells within tumour stroma promote breast cancer metastasis. Nature.

[B14] Hermann PC, Huber SL, Herrier T, Aicher A, Ellwart J, Guba M, Bruns CJ, Heeschen C (2007). Distinct populations of cancer stem cells determine tumor growth and metastatic activity in human pancreatic cancer. Cell Stem Cell.

[B15] Rak J (1989). Possible role of tumour stem-end cell cooperation in metastasis. Med Hypotheses.

[B16] Barnhart BC, Simon MC (2007). Metastasis and stem cell pathways. Cancer Metastasis Rev.

[B17] Radulescu RT (2007). Oncoprotein metastasis disjoined.. http://arxiv.org/abs/0712.2981.

[B18] Radulescu RT (2006). Peptide strings in detail: first paradigm for the theory of everything (TOE). Pioneer.

[B19] Radulescu RT (2007). Across and beyond the cell are peptide strings.. http://arxiv.org/abs/0711.0202.

[B20] Radulescu RT (2005). From particle biology to protein and peptide strings: a new perception of life at the nanoscale. Logical Biology.

[B21] Radulescu RT (2005). Invisible field beyond visible cells: It is time to jump over a "Berlin Wall" in cancer research. Logical Biology.

[B22] Radulescu RT (2003). Particle biology: at the interface between physics and metabolism. Logical Biology.

[B23] Gunlusoy B, Arslan M, Selek E, Sayhan HS, Minareci S, Cicek S (2004). A case report: renal metastasis of prostate cancer. Int Urol Nephrol.

[B24] Kleinmann N, Mor Y, Laufer M, Duvdevani M, Fridman E, Ramon J (2005). A solitary metastasis of breast cancer to the urinary bladder. Breast J.

[B25] Trastour C, Rahili A, Avallone S, Karimdjee BS, Chevallier A, Bongain A, Benchimol D (2007). Metastasis to the uterine cervix from a rectal cancer. Eur J Obstet Gynecol Reprod Biol.

[B26] Zagha RM, Hamawy KJ (2007). Solitary breast cancer metastasis to the bladder: an unusual occurrence. Urol Oncol.

[B27] Wood LD, Parsons DW, Jones S, Lin J, Sjoblom T, Leary RJ, Shen D, Boca SM, Barber T, Ptak J (2007). The Genomic Landscapes of Human Breast and Colorectal Cancers. Science.

[B28] Fearon ER, Vogelstein B (1990). A genetic model for colorectal tumorigenesis. Cell.

[B29] Kuukasjarvi T, Karhu R, Tanner M, Kahkonen M, Schaffer A, Nupponen N, Pennanen S, Kallioniemi A, Kallioniemi OP, Isola J (1997). Genetic heterogeneity and clonal evolution underlying development of asynchronous metastasis in human breast cancer. Cancer Res.

[B30] Pandis N, Teixeira MR, Adeyinka A, Rizou H, Bardi G, Mertens F, Andersen JA, Bondeson L, Sfikas K, Qvist H (1998). Cytogenetic comparison of primary tumors and lymph node metastases in breast cancer patients. Genes Chromosomes Cancer.

[B31] Klein CA, Blankenstein TJ, Schmidt-Kittler O, Petronio M, Polzer B, Stoecklein NH, Riethmuller G (2002). Genetic heterogeneity of single disseminated tumour cells in minimal residual cancer. Lancet.

[B32] Gray JW (2003). Evidence emerges for early metastasis and parallel evolution of primary and metastatic tumors. Cancer Cell.

[B33] Schmidt-Kittler O, Ragg T, Daskalakis A, Granzow M, Ahr A, Blankenstein TJ, Kaufmann M, Diebold J, Arnholdt H, Muller P (2003). From latent disseminated cells to overt metastasis: genetic analysis of systemic breast cancer progression. Proc Natl Acad Sci USA.

[B34] Solakoglu O, Maierhofer C, Lahr G, Breit E, Scheunemann P, Heumos I, Pichlmeier U, Schlimok G, Oberneder R, Kollermann MW (2002). Heterogeneous proliferative potential of occult metastatic cells in bone marrow of patients with solid epithelial tumors. Proc Natl Acad Sci USA.

[B35] Pantel K, Brakenhoff RH (2004). Dissecting the metastatic cascade. Nat Rev Cancer.

[B36] Radulescu RT (2003). Oncoprotein-induced "conformational mutation" of a tumor suppressor as an early event in oncogenesis: a novel concept. Logical Biology.

[B37] Liu SV (2005). A theoretical framework for understanding biotic aging from molecule to organism in multicellular life. Logical Biology.

[B38] Meza R, Luebeck EG, Moolgavkar SH (2005). Gestational mutations and carcinogenesis. Math Biosci.

[B39] Knudson AG (1971). Mutation and cancer: statistical study of retinoblastoma. Proc Natl Acad Sci USA.

[B40] Vaidya JS (2007). An alternative model of cancer cell growth and metastasis. Int J Surg.

[B41] Stingl J, Caldas C (2007). Molecular heterogeneity of breast carcinomas and the cancer stem cell hypothesis. Nat Rev Cancer.

[B42] Bernards R, Weinberg RA (2002). A progression puzzle. Nature.

[B43] van 't Veer LJ, Dai H, van de Vijver MJ, He YD, Hart AA, Bernards R, Friend SH (2003). Expression profiling predicts outcome in breast cancer. Breast Cancer Res.

[B44] van 't Veer LJ, Dai H, van de Vijver MJ, He YD, Hart AA, Mao M, Peterse HL, van der Kooy K, Marton MJ, Witteveen AT (2002). Gene expression profiling predicts clinical outcome of breast cancer. Nature.

[B45] Takahashi K, Kohno T, Matsumoto S, Nakanishi Y, Arai Y, Yamamoto S, Fujiwara T, Tanaka N, Yokota J (2007). Clonal and parallel evolution of primary lung cancers and their metastases revealed by molecular dissection of cancer cells. Clin Cancer Res.

[B46] Liu SV (2005). Linking DNA aging with cell aging and combining genetics with epigenetics. Logical Biology.

[B47] Liu SV (2005). "Cellular senescence": What does it really mean?. Logical Biology.

[B48] Liu SV (1999). Tracking bacterial growth in liquid media and a new bacterial life model. Science in China (Series C: Life Science) (English).

[B49] Liu SV (2006). Revisit semi-conservative DNA replication and immortal DNA strand hypothesis. Logical Biology.

[B50] Liu SV (2006). Towards a deep understanding of the fundamental and universal mechanism of biotic aging. 3rd International Conference on Functional Genomics of Ageing March 29th – April 1st Palermo, Sicily, Italy.

[B51] Liu SV (2006). Evolution: an integrated theory – Criticisms on Darwinism – Fifteen years ago. Pioneer.

[B52] Bazykin GA, Kondrashov FA, Brudno M, Poliakov A, Dubchak I, Kondrashov AS (2007). Extensive parallelism in protein evolution. Biol Direct.

[B53] Liu SV (2006). Hard to swallow: Oncogenic "pills" for cancer?. Logical Biology.

[B54] Aguirre-Ghiso JA (2007). Models, mechanisms and clinical evidence for cancer dormancy. Nat Rev Cancer.

[B55] Dani DN, Sainis JK (2007). Modularity: a new perspective in biology. Indian J Biochem Biophys.

[B56] Tamames J, Moya A, Valencia A (2007). Modular organization in the reductive evolution of protein-protein interaction networks. Genome Biol.

[B57] He L, He X, Lim LP, de Stanchina E, Xuan Z, Liang Y, Xue W, Zender L, Magnus J, Ridzon D (2007). A microRNA component of the p53 tumour suppressor network. Nature.

[B58] He L, He X, Lowe SW, Hannon GJ (2007). microRNAs join the p53 network – another piece in the tumour-suppression puzzle. Nat Rev Cancer.

[B59] Duelli D, Lazebnik Y (2007). Cell-to-cell fusion as a link between viruses and cancer. Nat Rev Cancer.

[B60] Duelli DM, Padilla-Nash HM, Berman D, Murphy KM, Ried T, Lazebnik Y (2007). A virus causes cancer by inducing massive chromosomal instability through cell fusion. Curr Biol.

[B61] Ince TA, Richardson AL, Bell GW, Saitoh M, Godar S, Karnoub AE, Iglehart JD, Weinberg RA (2007). Transformation of different human breast epithelial cell types leads to distinct tumor phenotypes. Cancer Cell.

[B62] Lin J, Gan CM, Zhang X, Jones S, Sjoblom T, Wood LD, Parsons DW, Papadopoulos N, Kinzler KW, Vogelstein B (2007). A multidimensional analysis of genes mutated in breast and colorectal cancers. Genome Res.

[B63] Ochsenkuhn T, Bayerdorffer E, Meining A, Spath L, Mannes GA, Wiebecke B, Eiermann W, Sackmann M, Goke B (2005). Increased prevalence of colorectal adenomas in women with breast cancer. Digestion.

[B64] Kmet LM, Cook LS, Weiss NS, Schwartz SM, White E (2003). Risk factors for colorectal cancer following breast cancer. Breast Cancer Res Treat.

[B65] Newschaffer CJ, Topham A, Herzberg T, Weiner S, Weinberg DS (2001). Risk of colorectal cancer after breast cancer. Lancet.

[B66] Vasen HF, Morreau H, Nortier JW (2001). Is breast cancer part of the tumor spectrum of hereditary nonpolyposis colorectal cancer?. Am J Hum Genet.

[B67] Cook LS, Weiss NS, Pharris-Ciurej N, Schwartz SM, White E (2001). Colorectal cancer following tamoxifen therapy for breast cancer (United States). Cancer Causes Control.

[B68] Lin KM, Ternent CA, Adams DR, Thorson AG, Blatchford GJ, Christensen MA, Watson P, Lynch HT (1999). Colorectal cancer in hereditary breast cancer kindreds. Dis Colon Rectum.

[B69] Wu CS, Tung SY, Chen PC, Kuo YC, Wang CY (1997). Colorectal adenoma in patients with a history of breast cancer: a prospective study in Taiwan. Int J Clin Pract.

[B70] Sankila R, Hakulinen T (1998). Survival of patients with colorectal carcinoma: effect of prior breast cancer. J Natl Cancer Inst.

[B71] Boyd J, Rhei E, Federici MG, Borgen PI, Watson P, Franklin B, Karr B, Lynch J, Lemon SJ, Lynch HT (1999). Male breast cancer in the hereditary nonpolyposis colorectal cancer syndrome. Breast Cancer Res Treat.

[B72] Schoen RE, Weissfeld JL, Kuller LH (1994). Are women with breast, endometrial, or ovarian cancer at increased risk for colorectal cancer?. Am J Gastroenterol.

[B73] Yilmaz OH, Valdez R, Theisen BK, Guo W, Ferguson DO, Wu H, Morrison SJ (2006). Pten dependence distinguishes haematopoietic stem cells from leukaemia-initiating cells. Nature.

[B74] Howell MA (1976). The association between colorectal cancer and breast cancer. J Chronic Dis.

[B75] Agarwal N, Ulahannan MJ, Mandile MA, Cayten CG, Pitchumoni CS (1986). Increased risk of colorectal cancer following breast cancer. Ann Surg.

[B76] Toma S, Giacchero A, Bonelli L, Graziani A, De Lorenzi R, Aste H (1987). Association between breast and colorectal cancer in a sample of surgical patients. Eur J Surg Oncol.

[B77] Neugut AI, Murray TI, Lee WC, Robinson E (1991). The association of breast cancer and colorectal cancer in men. An analysis of surveillance, epidemiology, and end results program data. Cancer.

[B78] Murray TI, Neugut AI, Garbowski GC, Waye JD, Forde KA, Treat MR (1992). Relationship between breast cancer and colorectal adenomatous polyps. A case-control study. Cancer.

[B79] Neugut AI (1993). Breast cancer and colorectal neoplasia: double jeopardy or not?. Am J Gastroenterol.

[B80] Garrett CT, Liscia DS, Nasim S, Ferreira-Gonzalez A (1995). Genetics of colorectal and breast cancer. Clin Lab Med.

[B81] Maione S, Giunta A, Agozzino L (1985). Unusual intracardiac metastasis of a testicular embryonal carcinoma. Int J Cardiol.

[B82] Pickuth D, Eeles R, Mason M, Pumphrey C, Goldstraw P, Horwich A (1992). Intracardiac metastases from germ cell tumours – an unusual but important site of metastasis. Br J Radiol.

[B83] Nicolson GL (1988). Organ specificity of tumor metastasis: role of preferential adhesion, invasion and growth of malignant cells at specific secondary sites. Cancer Metastasis Rev.

[B84] van Oijen MG, Leppers Vd Straat FG, Tilanus MG, Slootweg PJ (2000). The origins of multiple squamous cell carcinomas in the aerodigestive tract. Cancer.

[B85] Dinjens WN, van der Burg ME, Chadha S, Sleddens HF, Burger CW, Ewing PC (2003). Clinical importance of molecular determinations in gynecologic patients with multiple tumors. Cancer.

[B86] el-Naggar AK, Hurr K, Batsakis JG, Luna MA, Goepfert H, Huff V (1995). Sequential loss of heterozygosity at microsatellite motifs in preinvasive and invasive head and neck squamous carcinoma. Cancer Res.

[B87] el-Naggar AK, Lai S, Luna MA, Zhou XD, Weber RS, Goepfert H, Batsakis JG (1995). Sequential p53 mutation analysis of pre-invasive and invasive head and neck squamous carcinoma. Int J Cancer.

[B88] Cianfrigkia F, Di Gregorio DA, Manieri A (1999). Multiple primary tumors in patients with oral squamous cell carcinoma. Oral Oncol.

[B89] Razin A, Riggs AD (1980). DNA methylation and gene function. Science.

[B90] Chung KY, Mukhopadhyay T, Kim J, Casson A, Ro JY, Goepfert H, Hong WK, Roth JA (1993). Discordant p53 gene mutations in primary head and neck cancers and corresponding second primary cancers of the upper aerodigestive tract. Cancer Res.

[B91] Slaughter DP, Southwick HW, Smejkal W (1953). Field cancerization in oral stratified squamous epithelium; clinical implications of multicentric origin. Cancer.

[B92] Partridge M, Emilion G, Pateromichelakis S, Phillips E, Langdon J (1997). Field cancerisation of the oral cavity: comparison of the spectrum of molecular alterations in cases presenting with both dysplastic and malignant lesions. Oral Oncol.

[B93] van Oijen MG, Slootweg PJ (2000). Oral field cancerization: carcinogen-induced independent events or micrometastatic deposits?. Cancer Epidemiol Biomarkers Prev.

[B94] Odrazka K, Petera J, Zouhar M, Vosmik M, Vaculikova M, Dolezel M, Kohlova T, Filip S, Ceral J, Hobza V (2005). Clinical results of intensity-modulated radiation therapy (IMRT) for tumors of the head and neck region. Neoplasma.

[B95] Odrazka K, Vanasek J, Vaculikova M, Stejskal J, Filip S (2000). The role of chemotherapy in prostate cancer. Minireview. Neoplasma.

[B96] Koebel CM, Vermi W, Swann JB, Zerafa N, Rodig SJ, Old LJ, Smyth MJ, Schreiber RD (2007). Adaptive immunity maintains occult cancer in an equilibrium state. Nature.

[B97] Melief CJ (2007). Cancer: immune pact with the enemy. Nature.

[B98] Minn AJ, Gupta GP, Siegel PM, Bos PD, Shu W, Giri DD, Viale A, Olshen AB, Gerald WL, Massague J (2005). Genes that mediate breast cancer metastasis to lung. Nature.

[B99] Smith MC, Luker KE, Garbow JR, Prior JL, Jackson E, Piwnica-Worms D, Luker GD (2004). CXCR4 regulates growth of both primary and metastatic breast cancer. Cancer Res.

[B100] Kang Y, He W, Tulley S, Gupta GP, Serganova I, Chen CR, Manova-Todorova K, Blasberg R, Gerald WL, Massague J (2005). Breast cancer bone metastasis mediated by the Smad tumor suppressor pathway. Proc Natl Acad Sci USA.

[B101] Trent JM, Touchman JW (2007). CANCER: The Gene Topography of Cancer. Science.

[B102] Fidler IJ, Yano S, Zhang RD, Fujimaki T, Bucana CD (2002). The seed and soil hypothesis: vascularisation and brain metastases. Lancet Oncol.

[B103] Filip S, Mokry J, Karbanova J, Vavrova J, English D (2004). Local environmental factors determine hematopoietic differentiation of neural stem cells. Stem Cells Dev.

[B104] Ricci-Vitiani L, Lombardi DG, Pilozzi E, Biffoni M, Todaro M, Peschle C, De Maria R (2007). Identification and expansion of human colon-cancer-initiating cells. Nature.

[B105] O'Brien CA, Pollett A, Gallinger S, Dick JE (2007). A human colon cancer cell capable of initiating tumour growth in immunodeficient mice. Nature.

[B106] Husemann Y, Geigl JB, Schubert F, Musiani P, Meyer M, Burghart E, Forni G, Eils R, Fehm T, Riethmuller G, Klein CA (2008). Systemic spread is an early step in breast cancer. Cancer Cell.

[B107] Radulescu RT (2005). Normal cells first: a possible Copernican turn in cancer therapy. Logical Biology.

[B108] Radulescu RT (2005). Disconnecting cancer cybernetics through a dual anti-nucleocrine strategy: towards an anticipatory therapy of malignant disease. Logical Biology.

[B109] Radulescu RT (2008). Going beyond the genetic view of cancer. Proc Natl Acad Sci USA.

[B110] Filip S (2006). The phenomenon of stem cell plasticity: biological or physiological problems?. Stem Cells Dev.

[B111] Filip S, Mokry J, English D (2006). Stem cell plasticity and carcinogenesis. Neoplasma.

[B112] Filip S, Mokry J, English D, Vojacek J (2005). Stem cell plasticity and issues of stem cell therapy. Folia Biol (Praha).

[B113] Simpson PT, Da Silva LM, Lakhani SR (2007). In situ carcinoma – can we predict which patient will come back with a recurrence?. Cancer Cell.

[B114] van Noort V, Snel B, Huynen MA (2007). Exploration of the omics evidence landscape: adding qualitative labels to predicted protein-protein interactions. Genome Biol.

[B115] Brandt B, Griwatz C, Brinkmann O, Zanker KS (1997). Circulating prostate-specific antigen/CD14-double-positive cells: a biomarker indicating low risk for hematogeneous metastasis of prostate cancer. J Natl Cancer Inst.

[B116] Yoshikawa R, Yanagi H, Shen CS, Fujiwara Y, Noda M, Yagyu T, Gega M, Oshima T, Yamamura T, Okamura H (2006). ECA39 is a novel distant metastasis-related biomarker in colorectal cancer. World J Gastroenterol.

